# A Comparative Evaluation of Four Bioinformatic Tools for Identifying HIV-1 *pol* Drug Resistance Mutations Using Illumina MiSeq Data

**DOI:** 10.3390/biology15050438

**Published:** 2026-03-07

**Authors:** Ogestelli Fabia Lee, Chun Kiat Lee

**Affiliations:** 1 Clinical Research & Innovation Office, National Healthcare Group, Singapore 308433, Singapore; ogestellifabialee@gmail.com; 2 Department of Laboratory Medicine, National University Health System, Singapore 119074, Singapore

**Keywords:** HIV-1 drug resistance, next-generation sequencing, bioinformatics, low-abundance mutations, genotype-to-phenotype algorithms

## Abstract

The transition from Sanger to next-generation sequencing (NGS) for HIV-1 drug resistance testing offers enhanced sensitivity but also introduces bioinformatic variability. This study evaluated four strategies: the commercial Exatype platform, the academic Stanford HIVdb-NGS, the open-source Quasitools (HyDRA) suite, and a custom de novo assembly workflow, iLunaR. Using 85 clinical HIV-1 *pol* MiSeq sequencing datasets, concordance was assessed at a 2% mutation detection threshold. A majority consensus standard defined true presence if a mutation was detected by at least three pipelines and supported by Sanger sequencing. While the datasets were successfully processed by all pipelines, discordances emerged in detecting low-abundance mutations and a specific case of structural mutation. iLunaR achieved perfect agreement (Cohen’s kappa = 1.000; 95% CI: 1.000–1.000). Quasitools demonstrated the lowest agreement (Cohen’s kappa = 0.901; 95% CI: 0.807–0.995) due to consistent reporting of mutations at lower abundance levels and aligner-induced reference bias misclassifying a deletion as a point mutation. Exatype (Cohen’s kappa = 0.951; 95% CI: 0.884–1.000) and Stanford (Cohen’s kappa = 0.926; 95% CI: 0.846–1.000) exhibited specific failures, including an omitted integrase mutation and codon translation errors, respectively. These findings confirm that bioinformatic algorithm choice remains a critical clinical variable despite NGS advancements in HIV-1 drug resistance testing.

## Simple Summary

Successful treatment of the human immunodeficiency virus depends on identifying genetic changes that make antiretroviral therapy medications ineffective. For decades, a traditional laboratory method called Sanger sequencing was the gold standard, but it often fails to detect rare, resistant versions of the virus that exist at low levels. Laboratories are now transitioning to next-generation sequencing, which is much more sensitive but relies on complex analysis workflows that can produce inconsistent results. This study addressed the variability in bioinformatic tools used to identify these changes. We compared four bioinformatic tools to determine which best identifies these low-abundance mutations using eighty-five next-generation sequencing datasets. We found that while most tools work well for common mutations, they struggle with low-abundance mutations or complex genetic structures. A custom approach that reconstructs the viral genetic code from scratch, known as de novo assembly, was the most accurate. Other tools missed critical resistance markers or misclassified genetic changes. These findings prove that the choice of bioinformatic tool is a vital part of medical care. By using more precise tools, laboratories can provide more reliable reports, ensuring patients receive the most effective treatments.

## 1. Introduction

The Joint United Nations Programme on HIV/AIDS (UNAIDS) has established the 95-95-95 targets for 2025, aiming for 95% of people living with HIV to know their status, 95% of those diagnosed to receive antiretroviral therapy (ART), and 95% of those on ART to achieve viral suppression [1]. Success in these targets depends on the timely detection of HIV-1 drug resistance (HIVDR) as the therapeutic landscape shifts toward integrase strand transfer inhibitors (INSTIs) and long-acting formulations [2,3]. HIV-1 is characterized by extreme genetic heterogeneity, with Group M alone comprising 10 subtypes and over 158 circulating recombinant forms (CRFs), complicating both diagnostic accuracy and resistance surveillance [1].

For decades, Sanger sequencing (SS) served as the gold standard, but it fails to detect mutations below a 20% frequency threshold. This creates a “blind spot” for low-abundance mutations (LAMs) persisting between 1% and 20% [4]. Evidence indicates that LAMs, such as the non-nucleoside reverse transcriptase inhibitor (NNRTI)-resistant K103N or Y181C, are independent predictors of virological failure [3]. Furthermore, “archived” mutations, such as the thymidine analog mutations (TAMs) selected by prior zidovudine exposure, may wane to levels undetectable by SS but persist as LAMs, ready to rebound under selective pressure [3]. Consequently, clinical laboratories are transitioning to next-generation sequencing (NGS) platforms that offer deep coverage capable of detecting mutations at frequencies as low as 1%. While NGS solves the sensitivity problem, it creates a new challenge regarding bioinformatics [4].

Transforming millions of short reads into clinical reports requires sophisticated algorithms for read alignment and variant calling. Currently, no single standardized pipeline exists, and laboratories must choose between commercial black-box solutions, academic web servers, and open-source tools. In this study, we evaluated four distinct bioinformatic strategies: Exatype NGS HIV (Hyrax Biosciences, Cape Town, South Africa), a commercial solution utilizing dynamic reference mapping; Stanford HIVdb-NGS, the academic standard employing a Minimap2/PostAlign strategy; Quasitools (HyDRA), an open-source suite using Bowtie2 for reference-based mapping; and iLunaR, a custom workflow employing de novo assembly to minimize reference bias.

## 2. Materials and Methods

### 2.1. Sequencing Dataset

We analyzed 85 de-identified HIV-1 *pol* FASTQ datasets derived from plasma samples of patients attending the National University Hospital, Singapore. The sequences spanned the protease (PR), reverse transcriptase (RT), and integrase (IN) regions. Sequencing was performed using an amplicon-based strategy on the Illumina MiSeq platform (Illumina Inc., San Diego, CA, USA) with paired-end chemistry (2 × 150), as previously described [4]. Matched population-based Sanger sequencing (SS) data served as the baseline.

### 2.2. Bioinformatics Pipelines

#### 2.2.1. Exatype NGS HIV

The Exatype NGS HIV version 0.9.2.17 (Hyrax Biosciences, Cape Town, South Africa) is a commercial, cloud-based bioinformatics platform designed for the automated processing of HIV-1 sequencing data. Alignment is performed using the Rapid Amplicon Mapping in Codon Space (RAMICS) logic [5], which utilizes a dynamic reference mapping engine and a codon-aware strategy to penalize non-biological frameshifts while preserving genuine reading frame disruptions. Variant calling using the RAMICS logic models error distribution to distinguish technical sequencing errors, such as homopolymer-related artifacts, from true biological LAMs. For this study, the pipeline was used with its default configuration, with the exception of the mutation detection threshold (MDT), which was manually preset to report mutations at a 2% prevalence threshold. Drug resistance interpretation was automatically integrated through the Stanford HIV Drug Resistance Database algorithm.

#### 2.2.2. Stanford University HIVdb-NGS

The Stanford University HIVdb-NGS tool (beta) is a web-based service that processes raw FASTQ files into standardized codon frequency tables (Stanford University; https://hivdb.stanford.edu/hivdb/by-reads/; accessed on 20 December 2025). Preprocessing involved trimming adapters and low-quality bases using fastp. Reads were aligned to HXB2 using Minimap2, followed by the PostAlign algorithm for codon-aware refinement of indels.

We applied a minimum read depth of 250 and an MDT of 1% for this analysis. Notably, this threshold was specifically lowered to capture mutations at the 2% level that were sporadically omitted when the MDT was set to the default 2%. Furthermore, the nucleotide mixture threshold (NMT) option was explicitly disabled. Disabling NMT was essential, as internal validation revealed that several clinically relevant mutations were inadvertently filtered when the option was active.

#### 2.2.3. Quasitools (HyDRA)

Quasitools version 0.7.0 (quasitools; https://phac-nml.github.io/quasitools/; accessed on 4 June 2025) is an open-source bioinformatics suite developed by the National Microbiology Laboratory at the Public Health Agency of Canada. This study employed the HyDRA (Hypermutation Detection and Read Alignment) workflow, which aligns reads to an annotated HXB2 *pol* reference sequence using Bowtie2 (version 2.5.4). The pipeline incorporates a specialized filtering step to identify and remove sequences exhibiting APOBEC3G/F-mediated hypermutation. Following the exclusion of hypermutated reads, amino acid mutations were quantified relative to the HXB2 reading frame using a minimum coverage depth of 250 reads and an MDT of 2%. For the purposes of this evaluation, the default parameters were utilized for Bowtie2 read alignment and hypermutation filtering.

#### 2.2.4. iLunaR

The iLunaR pipeline is a custom bioinformatics workflow developed to mitigate reference bias through an “assembly-first” approach. Built within an Ubuntu 23.04 (Lunar Lobster) environment, the pipeline integrates Bash shell scripting for data processing and R for the statistical calculation of mutation abundance. A defining characteristic of iLunaR is its organism-agnostic architecture; unlike reference-constrained pipelines developed solely for HIV-1, iLunaR provides a universal workflow adaptable to highly divergent pathogens, somatic oncology, and complex human genomic analysis. This strategy is particularly valuable for detecting structural mutations that standard alignment pipelines often overlook [6,7,8].

Briefly, raw paired-end reads were subjected to quality trimming and adapter removal using Trimmomatic (version 0.39) [9]. Adapters were removed using the ILLUMINACLIP:NexteraPE-PE.fa:2:30:10:8:true parameter, and reads were further refined using the LEADING:30 and TRAILING:30 flags to exclude bases with a quality score below Q30. The processed reads were then subjected to de novo assembly using MEGAHIT (version 1.2.9) to mitigate reference bias [10]. Following iterative testing to determine the optimal parameters for MiSeq amplicon reconstruction, the minimum k-mer size was set to 19, while all other assembly parameters were maintained at their default settings. Resulting assemblies were queried against the HIV-1 HXB2 reference (GenBank accession K03455.1) using SSEARCH36 (version 36.3.8i), and the contig with the highest similarity score is extracted as a sample-specific reference [11]. Paired-end reads are subsequently remapped to this sample-specific reference using the BWA-SW algorithm (version 0.7.17-r1188), and alignments are processed by SAMtools (version 1.16.1) and BEDTools (version 2.30.0) to generate pileup and coverage data [12,13,14]. To ensure high sensitivity in capturing diverse viral populations, the BWA-SW parameters were specifically configured to track a higher number of potential hits. The Z-best value (-z) was increased to 10, allowing the aligner to explore multiple seeding candidates before finalizing the local alignment. This was paired with the use of 8 threads (-t 8) to manage the increased computational load required by this higher sensitivity setting. All other parameters were maintained at their default values. Rapid variant calling is achieved using a custom script utilizing the GNU Parallel tool (version 20221122) for multi-threaded, direct parsing of the raw mpileup string, followed by an R script to compute the final mutation abundance and generate the consensus sequence at an MDT of 2%.

### 2.3. Drug Resistance Mutation Interpretation and Consensus Definition

Drug resistance mutation (DRM) interpretation was conducted using the Stanford HIV Drug Resistance Database Version 9.8 (Stanford University; https://hivdb.stanford.edu/hivdb/by-patterns/; accessed on 20 December 2025) to identify clinically relevant mutations. To establish a reference standard for assay performance, a majority consensus standard was defined for each sample. A mutation was considered truly present if it was detected by at least three of the four NGS pipelines at a frequency ≥ 2% or supported by SS.

### 2.4. Statistical Analysis

For accuracy, we calculated positive percentage agreement (PPA), negative percentage agreement (NPA), and Cohen’s kappa coefficient using 95% confidence intervals (CIs). All statistical analyses were performed using the R statistical language version 3.6.0 (R Foundation for Statistical Computing, Vienna, Austria; https://www.R-project.org/).

## 3. Results

### 3.1. Concordance and Sanger Comparison

All four pipelines successfully processed 100% of the datasets (*n* = 85). A summary of the sequencing metrics, including mean read count and quality scores (Q30), is presented in Table 1. A spectrum of DRMs was identified, and the consensus mutations are detailed in Table 2. High inter-pipeline concordance was observed, with 76 of 85 samples showing identical drug resistance profiles at 2% MDT, representing an overall concordance rate of 89.4% (95% CI: 81.10–94.30%). Discrepancies were identified in 9 samples (10.6%), driven exclusively by LAMs that remained undetected by SS.

### 3.2. Cohen’s Kappa, Positive Percent Agreement, and Negative Percent Agreement

Diagnostic performance was benchmarked against the majority consensus standard, which identified 35 positive and 50 negative DRM results. Table 3 provides the detailed statistical summary. While all pipelines demonstrated perfect specificity (NPA = 100.00%; 95% CI: 92.90–100.00%), sensitivity varied across mapping-based strategies. The assembly-first iLunaR pipeline achieved perfect diagnostic agreement (Cohen’s kappa = 1.000; PPA = 100.00%). In contrast, a gradient of declining sensitivity was observed for the mapping-dependent tools, with Exatype (PPA = 94.30%; Cohen’s kappa = 0.951), Stanford (PPA = 91.40%; Cohen’s kappa = 0.926), and Quasitools (PPA = 88.60%; Cohen’s kappa = 0.901) exhibiting increasing rates of false-negative reports.

### 3.3. Discrepancy Analysis

Quasitools missed consensus mutations in four samples (S6, S7, S9, and S43), all within the RT region. For sample S6, the H221Y mutation was quantified at ~2.0% by other tools but not detected by Quasitools even at 1% MDT. Similarly, the T215S mutation was quantified at ~4.0% for sample S7 by other tools but reported at only 1.74% by Quasitools. In sample S9, the H221Y mutation was again quantified at ~2.0% by other tools but reported at only 1.92% by Quasitools. Finally, for sample S43, Quasitools misreported a structural mutation (T69Tdel) as a point mutation (T69G) with an 8.7% frequency.

The Stanford HIVdb-NGS pipeline failed to report consensus mutations in three samples (S25, S34, and S38), all within the RT region. In sample S25, Stanford misreported the D67N mutation as D67E. In sample S34, the M230L mutation was quantified at ~2.0% by other tools but was not detected by Stanford, even at 1% MDT. In sample S38, the Y181C mutation was quantified at ~3.0% by other tools but reported at only 1.0% by Stanford.

**Figure 1:**
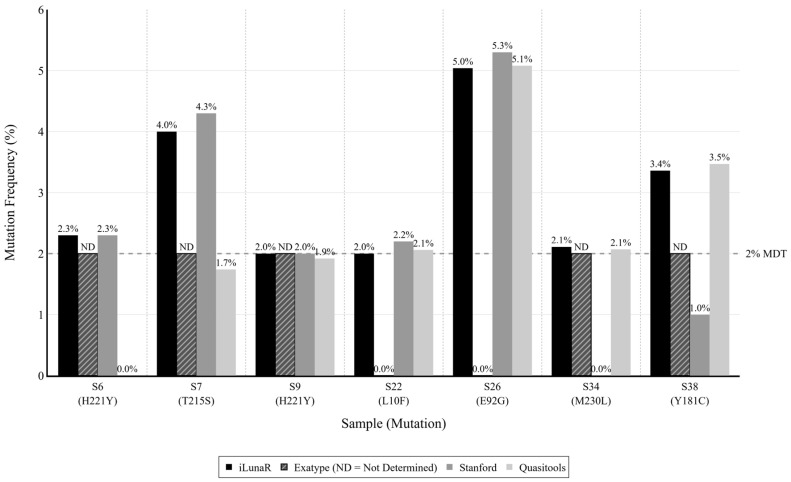
Comparison of reported mutation frequencies across four bioinformatic pipelines (iLunaR, Exatype, Stanford HIVdb, and Quasitools) for discordant low-abundance mutations. The dashed horizontal line represents the 2.0% mutation detection threshold (MDT). For Exatype, “ND” (not determined) indicates that the mutation was detected at the 2.0% prevalence threshold, but a specific frequency percentage was not reported by the pipeline.

Finally, the Exatype NGS HIV platform missed consensus mutations in two samples (S22 and S26) involving the PR and IN regions, respectively. In sample S22, the L10F mutation was quantified at ~2.0% by other tools but not detected by Exatype. The pipeline also failed to report a ~5.0% E92G mutation in sample S26, which was correctly identified by all other platforms. All discrepancies are summarized in Table 2. The distribution of mutation frequencies across discordant samples is depicted in Figure 1.

## 4. Discussion

As HIV-1 genotyping shifts toward NGS to meet the 95-95-95 targets, the bioinformatic pipeline becomes a critical clinical variable that dictates the sensitivity and reliability of resistance reporting [1]. This study demonstrates that while standard tools are robust for dominant viral populations, significant divergence occurs in the “grey zone” of LAMs between 1% and 5%.

### 4.1. Quasitools (HyDRA)

The systematic under-quantification of mutations by Quasitools serves as a cautionary tale regarding the application of rigid clinical thresholds. In our study, sample S7 contained the NRTI-revertant mutation T215S. T215S is a critical sentinel marker for archived thymidine analogue mutation (TAM) resistance, typically emerging when the virus reverts from the high-level resistance mutations T215Y or T215F in the absence of drug pressure. These revertants are highly stable and signify a virus that can rapidly re-evolve high-level zidovudine resistance upon re-exposure, potentially compromising future NRTI backbones [15,16]. While iLunaR, Stanford, and Exatype detected this at ~4%, Quasitools reported it at 1.74%, leading to a false-negative classification under a 2% MDT. Similarly, the NNRTI resistance mutation H221Y was missed in samples S6 and S9 due to this quantification dampening. H221Y is associated with decreased susceptibility to the second-generation NNRTI rilpivirine, and its presence as a LAM is a significant independent predictor of virological failure in patients initiating first-line ART [15]. Since missing these signals could lead to the prescription of compromised regimens, laboratories utilizing conservative algorithms like HyDRA should consider validating lower reporting thresholds to ensure these clinically impactful mutations are not overlooked.

The misclassification of the T69 deletion in sample S43 reveals the potential impact of reference bias on standard alignment algorithms. While Exatype, Stanford, and iLunaR successfully identified the deletion, Quasitools mislabeled it as a T69G point mutation. This error may be attributed to the Bowtie2 aligner used in Quasitools, which imposes high affine gap penalties, forcing reads containing the deletion to match the HXB2 reference as substitutions rather than opening a gap. The clinical impact of this misclassification is severe because the T69 deletion confers high-level resistance to almost all approved NRTIs [17]. Exatype employs a dynamic reference mapping approach that likely selects a reference sequence closer to the patient’s viral strain, thereby reducing the alignment penalty. Similarly, Stanford’s pipeline utilizes a specialized PostAlign step that performs codon-aware realignment to correctly place indels. iLunaR achieved the same correct result through de novo assembly, which physically reconstructs the deletion in the reference contig.

### 4.2. Exatype NGS HIV

The “black-box” nature of commercial platforms presents a distinct challenge for clinical validation and root-cause analysis of discrepancies. In our study, Exatype failed to report the ~2% L10F PR mutation in sample S22 and the ~5% E92G IN mutation in sample S26. L10F is a nonpolymorphic accessory mutation selected by lopinavir, darunavir, and atazanavir. Its presence reduces virological response to multiple protease inhibitors when combined with other mutations [15]. The omission of E92G is particularly concerning, as it reduces elvitegravir susceptibility by over 10-fold and is a primary resistance-associated mutation within the integrase catalytic core [15,18].

Additionally, unlike open-source tools, where parameters can be iteratively adjusted and re-run, Exatype operates on a credit-based pricing model where each analysis consumes a per-sample credit. This creates a significant financial barrier to optimization. For instance, if a laboratory needs to re-analyze a sample at a 1% detection threshold to investigate a potential false negative at 2%, they must expend an additional analysis credit. Furthermore, the automated clinical reports generated by Exatype do not provide the quantitative abundance levels for detected mutations, leaving clinicians and laboratory staff without the granular quantitative data necessary for troubleshooting.

### 4.3. Stanford University HIVdb-NGS

This study provides the first external validation of the Stanford HIVdb-NGS (beta) pipeline. Our findings indicate that specific configurations, lowering the MDT to 1% and disabling the NMT option, are necessary to capture clinically relevant DRMs reliably at a 2% MDT.

The misclassification of D67N as D67E in sample S25 identifies a specific translation logic vulnerability. Since asparagine (N) and glutamic acid (E) share partial homology, this is likely to represent a translation logic error in handling IUPAC ambiguity codes within the pipeline. While D67N is a classic type 2 TAM, D67E is a zidovudine-selected mutation that is often part of more complex resistance patterns [19]. This suggests that the internal IUPAC resolution logic for mixed populations requires further refinement to avoid erroneous genotypic susceptibility scoring. 

Additionally, the missed detection of M230L in sample S34 and the under-quantification of Y181C in sample S38 (1.0% vs. 3.0% in other tools) reveal a pattern of quantification dampening comparable to Quasitools. M230L confers high-level resistance to the entire NNRTI class, including the second-generation drug doravirine; such omissions have profound clinical consequences [15]. Y181C is a major NNRTI resistance mutation conferring high-level resistance to nevirapine and efavirenz and reduced susceptibility to rilpivirine and etravirine. Even as a LAM, Y181C has been shown to more than triple the risk of virological failure in adherent patients initiating efavirenz-based regimens [20,21]. These omissions underscore that the Stanford HIVdb-NGS (beta) pipeline can fail to capture high-impact LAMs without careful, manual parameter optimization.

### 4.4. Implications for the High-Sensitivity Era of Next-Generation Sequencing

As clinical practice increasingly relies on detecting LAMs to prevent virological failure, laboratories must prioritize pipelines that incorporate advanced strategies to resolve structural complexity while remaining vigilant against the quantification biases inherent in specific tools.

### 4.5. Limitations of the Study

A key limitation of the present study is that the use of de-identified clinical datasets precluded the correlation of detected LAMs with longitudinal clinical records or prior ART history. Future studies incorporating retrospective clinical data would be invaluable to further validate the positive predictive value of these bioinformatic pipelines in identifying treatment-experienced patients at risk of virological failure.

## 5. Conclusions

This comparative analysis reveals that bioinformatics is a critical clinical variable in modern HIV management. While current NGS pipelines exhibit high concordance for dominant resistance mutations, significant variability remains in the detection and quantification of low-abundance mutations and structural anomalies. Laboratories implementing HIV-1 NGS must carefully validate their chosen software against international standards, such as the Winnipeg Consensus [22], and remain cognizant of the specific limitations inherent to reference-based or proprietary “black-box” approaches. Finally, the failure of traditional Sanger sequencing to detect any of the discordant low-abundance mutations confirms its inadequacy for high-precision molecular surveillance. Adopting standardized, transparent, and assembly-aware bioinformatic frameworks is essential for ensuring the integrity of resistance reporting in the third decade of antiretroviral therapy. 

## Figures and Tables

**Table 1:** Summary of sequencing read count and quality score (*n* = 85).

Parameter	Mean	95% Confidence Interval
Raw Read Count	83,764	75,635–91,893
Quality Score (% of bases ≥ Q30)	96.37%	95.92–96.82%

**Table 2:** Consensus drug resistance mutations for positive clinical samples (*n* = 35).

Sample	PI DRM	NRTI DRM	NNRTI DRM	INSTI DRM	Remarks
S1	L33F	M41L, L74I, M184V, L210W, T215F, K219N	K103N, V108I, N348I	-	Concordant
S2	-	A62V, K65R, V75I, M184V	K103N, Y318F, N348I	-	Concordant
S3	-	-	V106I, Y181C, G190A	-	Concordant
S4	-	-	V179D	-	Concordant
S5	-	A62V, K65R, D67H, S68G, V75I, K219E	L100I, Y181I, G190A, H221Y	-	Concordant
S6	-	M184I	K103N, H221Y, Y318F	E157Q	Discordant (Missed H221Y by Quasitools)
S7	-	T215S	V106I	-	Discordant (Missed T215S by Quasitools)
S8	-	M41L, D67N, K70R, M184V, L210W, T215Y	A98G, K101E, Y181YC, G190A	-	Concordant
S9	-	M184V, M184I	K103N, Y188L, H221Y, P225H	-	Discordant (Missed H221Y by Quasitools)
S11	-	M184I, K219E	E138K, E138Q	-	Concordant
S12	M46I	A62V, K65R, D67H, S68G, V75I, K219E	L100I, Y181I, G190A, H221Y	-	Concordant
S13	-	M184V	K103R, V106M, V179D	-	Concordant
S17	N88D	-	-	-	Concordant
S18	-	K70Q	K103N, V106A, H221Y, P225H	-	Concordant
S20	-	D67N, K70E, M184I, M184V	K103N, V179E, P225H	-	Concordant
S21	-	D67N, K70R, M184V, K219E, K219N	K103N, V106I, V108I, L234I, Y318F	-	Concordant
S22	L10F	Y115F, M184V	V106I, Y181C, Y188L, H221Y	R263K	Discordant (Missed L10F by Exatype)
S23	-	M41L, K65R, S68N, L74I, M184V	K101H, K103N, G190A, P225H	-	Concordant
S24	-	L74I, M184V	K103N, V179E, P225H	-	Concordant
S25	-	M41L, D67N, K70R, M184V, K219Q	Y181C, H221Y, N348I	-	Discordant (D67N misreported as D67E by Stanford)
S26	-	-	K103N, G190A	E92G	Discordant (Missed E92G by Exatype)
S27	-	-	K103N	-	Concordant
S28	-	K219E	Y181C, G190A, H221Y	N155H	Concordant
S29	-	M184V	-	-	Concordant
S31	-	-	-	T97A	Concordant
S33	-	-	V179D	E157Q	Concordant
S34	-	L74I, M184V	K103N, V108I, P225H, M230L	-	Discordant (Missed M230L by Stanford)
S35	-	K70E, M184V	K101P, E138K	-	Concordant
S36	-	M184V	-	T97A, Y143R, N155H	Concordant
S38	-	M184V	Y181C	-	Discordant (Missed Y181C by Stanford)
S39	-	K70R, L74I, L74V, M184V, K219E	V108I, Y181C, M230L	-	Concordant
S40	-	K65R, S68G, V75I, M184V	K101E, V106I, V108I, V179D, Y181C, G190A, H221Y	-	Concordant
S41	-	M41L, K70Q, M184V	K103N	-	Concordant
S42	-	M184V	V106I, E138Q, H221Y, F227C	-	Concordant
S43	-	T69Tdel	K103N, Y181C, G190A	-	Discordant (T69Tdel misreported as T69G by Quasitools)

PI: protease inhibitor; NRTI: nucleoside reverse transcriptase inhibitor; NNRTI: non-nucleoside reverse transcriptase inhibitor; INSTI: integrase strand transfer inhibitor; DRM: drug resistance mutation.

**Table 3:** Cohen’s kappa, positive percent agreement, and negative percent agreement (*n* = 85).

Pipeline	TP	TN	FP	FN	PPA (%) [95% CI]	NPA (%) [95% CI]	Cohen’s Kappa [95% CI]
iLunaR	35	50	0	0	100.00 [90.00, 100.00]	100.00 [92.90, 100.00]	1.000 [1.000, 1.000]
Exatype	33	50	0	2	94.30 [80.80, 99.30]	100.00 [92.90, 100.00]	0.951 [0.884, 1.000]
Stanford	32	50	0	3	91.40 [76.90, 98.20]	100.00 [92.90, 100.00]	0.926 [0.846, 1.000]
Quasitools	31	50	0	4	88.60 [73.30, 96.80]	100.00 [92.90, 100.00]	0.901 [0.807, 0.995]

TP: true positive; TN: true negative; FP: false positive; FN: false negative; PPA: positive percent agreement; NPA: negative percent agreement; CI: confidence interval.

## Data Availability

The data presented in this study are available upon request from the corresponding author. The data are not publicly available due to privacy and ethical concerns.
